# Patterns in quality of life according to employment among the older adults: the Korean longitudinal study of aging (2008–2014)

**DOI:** 10.1186/s12889-018-5296-x

**Published:** 2018-03-20

**Authors:** Deulle Min, Eunhee Cho

**Affiliations:** 1 Yonsei University College of Nursing, Seoul, Republic of Korea; 2 Yonsei University College of Nursing & Mo-Im Kim Nursing Research Institute, Seoul, Republic of Korea; 3 Yonsei University College of Nursing, 615 Nursing Education Building, 50-1 Yonsei-ro, Seodaemoon-Gu, Seoul, 03722 Republic of Korea

**Keywords:** Quality of life, Employment for the elderly, Aging

## Abstract

**Background:**

Korea is becoming an aged society. Accordingly, it is very meaningful to investigate the impact of job retention on quality of life (QOL) for older adults. We aimed to understand the pattern of changes over time in QOL of older adults aged 65 years or older based on employment status using the national data, the Korean Longitudinal Study of Aging (KLoSA).

**Methods:**

Data from the KLoSA during 2008–2014 were used. QOL was measured with the question, “How is your overall quality of life when compared to that of your peers in the same age group?” A total of 526 older adults were selected from 2008, including 267 who retained their jobs without change (job retention group) and 259 who lost their jobs by 2014 (job loss group). In order to analyze the factors affecting the QOL between 2008 and 2014, linear mixed models were used.

**Results:**

The average age of participants was 70 years. In men, the QOL was significantly higher in the job retention group than the job loss group (β = 7.751, *p* < .001). According to time, the QOL in 2012 (β = − 3.805, *p* = .003) and 2014 (β = − 4.254, p < .001) was significantly lower than that in 2008. In addition, the groups of retention or loss of job showed a significant difference in the change in QOL over time; the QOL was significantly lower in 2010 (β = − 3.570, *p* = .027) and 2014 (β = − 5.604, *p* = .003).

**Conclusions:**

This study found that employment is an important factor affecting the QOL. In order to improve the QOL of the older adults, tailored programs are needed to understand the characteristics of the elderly and to create suitable jobs for them.

## Background

According to the Korean National Statistical Office, as of 2015, the number of adults aged 65 or older had grown to 13.2% of the total population, and is expected to reach 40% in 2060 [1]. In 2016, the average life expectancy was 79.3 years for men and 85.4 years for women [2], and elderly living alone accounted for 6.4% of all households in South Korea [1]. However, the poverty rate of the elderly in South Korea is the highest among the Organization for Economic Co-operation and Development (OECD) countries, at about four times the OECD average [3]. Until the Employment Promotion Act was amended in 2016, anyone employed by a public institution or any enterprise employing more than 300 workers in South Korea had to retire at the age of 58, regardless of personal preference [4]. After retirement, most of the older adults wish to continue working in order to support themselves and their families [1]. However, 67.3% of wage workers over 60 years of age can only find temporary or part-time work, which does not guarantee a stable income [5], and most of the occupations undertaken by older people involve physical labor; in many cases, the environment is poor, and they work in occupations that young people avoid. In reality, it is difficult to get a job [6]. The average income of older adults aged 66~ 75 is only 62.4% of the national average income, far lower than the average of 90.1% found in other OECD countries. Over the next 50 years, the old-age support ratio will increase by 350% in South Korea; this is expected to be the highest increase among OECD countries [3].

As the elderly population increases, the social burden of caring for them also increases. Additionally, this weakens a society’s growth potential due to the decline in the percentage of economically active population [7]. As an example, in South Korea, all citizens are eligible for National Health Insurance; premiums are levied according to economic conditions, and older adults pay a reduced fee. When they receive treatment in a hospital, they pay only 10% to 30% of the total cost [8]. Thus, due to the increase in the aging population, the cost of medical insurance for older adults in 2015 increased to 36.8% of total medical expenses, which is about 3.34 million won per year, roughly three times the average medical cost per person [1]. While seniors’ life satisfaction and financial welfare are seen as important, South Korea lacks the social security or welfare systems to solve the problems this population experiences [9]. The national unemployment rate is 4.2%, and the unemployment rate of youths aged 20 to 29 years is about 11.3% [10]. In such a situation, chronic diseases and disabilities among older people are the main determinants of the withdrawal of older workers from the labor market [11]. In addition, Korean society divides the roles of men and women based on the influence of Confucianism, which emphasizes a strong father who is the breadwinner and a wise mother and good wife who devotes herself to the family. For this reason, to date, it remains harder for women to obtain employment than it is for men [12]. Recently, the government has enacted legislation to promote the employment of the elderly by prohibiting discrimination based on age, and is trying to increase the number of older workers by changing the age of legal retirement from 58 to 60 years [12]. However, these efforts are insufficient, particularly when compared to measures such as the Dutch Parliament’s bill to increase the retirement age from 65 to 67 years [13].

Previous studies on older adults’ work and quality of life (QOL) have shown that cases of depression have become more severe in unemployed persons over 50 years in the United States and Europe since the 2008 global economic crisis [14], that the formation of good relationships and social participation improves the QOL of older adults [15], and that QOL is found to be better for older adults who are involved in continuous physical activity [16]. In addition, the overall QOL is better among elderly persons with an occupation [17], and elderly persons who work are more likely to have a better QOL with regard to self-esteem, family, interpersonal relationships, and economic status [18]. On the other hand, it has also been argued that QOL declines due to lack of leisure time when an older adult gets a new job [18]. Furthermore, previous research has found that job loss does not affect health status [19, 20]. The impact of work on health and QOL has not yet been clarified.

In this context, most studies in Korea have examined factors affecting employment or the desire of older adults to return to employment [3, 21–23], while few studies have explored the effect of elderly employment programs on the QOL of older adults, or the impact of employment on the QOL of older adults [9, 18]. However, to improve the lives of the elderly, who comprise an increasing proportion of the population, it is important to identify patterns of change of QOL over time and in relation to employment in Korean society.

The global economic crisis in 2008 affected the domestic economy. Therefore, it is important to analyze the effect of employment on the QOL of those who remained employed from 2008 to 2014, and those who were employed prior to 2008 but lost their job during the crisis. In this study, we analyzed the pattern of changes over time in the QOL of older adults aged 65 years or older who had a job in 2008, using national data from the Korean Longitudinal Study of Aging (KLoSA) [24]. The results of this study will ultimately identify the specific impact of employment on the QOL of older adults and contribute to future policy development regarding the elderly.

## Methods

### Data and subjects

We used data drawn from the Korean Longitudinal Study of Aging (KLoSA) [24]. This study was designed to enable further comparative studies between developed countries that have already conducted aging panel surveys, including the United States (Health and Retirement Survey, or HRS), the UK (English Longitudinal Survey of Aging, or ELSA), and Europe (Survey of Health, Aging and Retirement in Europe, or SHARE). The Korean Labor Institute conducted KLoSA, a collection of data on middle-aged and senior citizens aged 45 years and above, for the purpose of producing basic data to establish and implement social and economic policies. Beginning in 2006, the KLoSA focused mainly on the population, family, health status, employment, income and consumption, assets, subjective expectations, and QOL. Sampling was conducted by sorting the population surveyed in a given area and residential type according to the order of the administrative codes, and then extracting the assigned number by applying a systematic extraction method (the multistage and stratified sampling method). The survey method used for the KLoSA was the Computer-Aided Personal Interview. Because it was important to examine the subjects’ job histories throughout their lives, the interviewers used a calendar to help respondents recall their memories. In addition, we applied weighting to reflect the difference between the information used in the sample design and the information used at the time of the survey.

The sample for this study was selected as follows. Of the 8688 respondents surveyed in 2008, the survival panel of older adults aged 65 or older was 4037, of whom 868 answered “Yes” to the question “Are you currently working for income?” Of these, 183 were excluded from the study as family workers (those who work for their own families), and 116 were excluded because they did not respond to the three surveys conducted since 2008. The remaining 569 were divided into two groups: one group comprised those who had retained a job (*n* = 308), and the other those who had lost their job before 2014 (*n* = 261). Of those in the job retention group, 39 had jobs in 2008 and 2014, but were excluded because they lost or had changed their employment in 2010 and 2012, and two subjects in each group did not respond to the questions regarding family income in the previous year. Therefore, a total of 526 respondents were selected, including 267 elderly persons who had retained their job without change (the job retention group) and 259 elderly persons who had lost their job by 2014 (the job loss group). In terms of job loss, 92 elderly respondents lost their job between 2008 and 2010 (Class 1), 83 between 2010 and 2012 (Class 3), and 80 between 2012 and 2014 (Class 4). Only four elderly respondents lost their job between 2008 and 2010 but were able to find a new one between 2010 and 2012, only to lose it again between 2012 and 2014 (Class 2). The purpose of this study was to investigate the association between employment and QOL.

The KLoSA data are publicly available, and can be downloaded from the employment survey site with personal information removed. This study was conducted following the approval of the Yonsei University Health System Research Ethics Committee (Y-2017-0007).

### Variables

#### Dependent variable

QOL was measured using the question: “How is your overall quality of life when compared to that of your peers in the same age group?” Responses were provided on a scale from 0 to 100 points at intervals of 10 points; higher scores indicated a higher QOL.

#### Independent variables

The sociodemographic characteristics of the job retention group and job loss group included age, educational background, total household income in the previous year, and residential area. Age was regarded as a continuous variable, and educational background was classified into four groups: elementary school, middle school, high school, and college or post-graduate work. A respondent’s total household income in the previous year was considered a continuous variable, and residential areas were classified as large cities, small cities, or rural areas.

The variables for health behaviors and health status included subjective health status, number of chronic diseases, grip strength index, depression, regular exercise, current smoking, and current drinking. Subjective health status was reclassified as good, moderate, and bad, and the number of chronic diseases was the number of chronic diseases (e.g., hypertension, diabetes, cancer, chronic lung disease, cerebrovascular disease, psychiatric disease, and arthritis) a respondent suffered from; the score range was 0–7. The body grip strength index was measured by the interviewers using a dynamometer, and the mean value of the grip strengths for the right hand and the left hand were used; the possible range was 0 to 50 kg. Depression scores were measured using 10 items of the Center for Epidemiological Studies – Depression (CES-D) instrument, which originally consisted of 20 items. The CES-D includes questions regarding a respondent’s feelings and behaviors in the past week, recording “1” for a depressive symptom and “0” for otherwise. The values are then added together to create a new variable, the possible range of which is from 0 to 10, with higher scores indicating that a respondent is “more depressed.” Regular exercise, current smoking, and current drinking were measured with two response options: “Yes” and “No” [25].

The social support system included: 1) whether the respondent lived alone; 2) whether the respondent was married; 3) the number and regularity of meetings with close friends; 4) the number of leisure activities (the total number of trips, sightseeing, outings, movies, performances, and concerts) the respondent engaged in over the past year; and 5) the time spent on self-development measured by the total time spent on hobbies, skill development, and volunteering. Living alone and being married were assessed with responses of “Yes” or “No.” Meeting with close friends was further broken down into the following frequencies: almost daily, 1–4 times a month, 1–6 times a year, and not meeting. The number of leisure activities and time spent on self-development were regarded as continuous variables.

### Statistical analysis

In this study, the analyses were conducted using the PASW SPSS WIN 23.0 program as follows. Descriptive statistics were used, such as percentages and averages of demographic characteristics, health status, health behaviors, and social support systems. Furthermore, differences between the two groups in 2008 were analyzed using an independent t-test and chi-square test. The changes and the differences in the QOL between the groups in 2008 and 2014 were analyzed using mean and standard deviations, with an independent t-test. Linear mixed models were used to analyze the effects of employment between 2008 and 2014 on QOL.

## Results

Table 1 shows the demographic characteristics, health status and health behaviors, social support systems and interactions, and differences between the groups. In the case of men, 59.8% of the job retention group resided in rural areas (χ^2^ = 26.04, *p* < .001), and 9.6% were more likely to participate in regular meetings than were the men in the job loss group (*t* = 5.24, *p* = .022). However, exercising regularly was 15.1% more common in the job loss group compared to the retention group (*t* = 10.99, *p* = .001), and living alone was 4.2% higher in the job loss group (*t* = 4.07, *p* = .044). In the case of women, the average age of the job loss group was more than that of the job retention group (*t* = − 2.41, *p* = .017); over 90% of the female respondents had no educational qualifications at all. The number of chronic diseases was 1.42 times higher in the job loss group than the job retention group (*t* = − 2.16, *p* = .032) and the depression score was 1.29 points higher as well (*t* = − 2.91, *p* = .004). The job loss group had more than twice the leisure time (*t* = − 2.06, *p* = .042), and self-development time was found to be higher in the job loss group (*t* = − 2.08, *p* = .041) (Table 1).

**Table 1:** Comparison of characteristics of job retention and loss groups in 2008

Variables	Category	Men (*N* = 383)			Women (*N* = 143)		
		Retention (*N* = 204) *N* (%) or Mean±SD	Loss (*N* = 179) *N* (%) or Mean±SD	Differences t or χ^2^ (*p)*	Retention (*N* = 63) *N* (%) or Mean±SD	Loss (*N* = 80) (*N* = 80) *N* (%) or Mean±SD	Differences t or χ^2^ (*p)*
Demographic characteristics							
Age		70.05 ± 4.11	70.40 ± 4.60	−0.77 (.442)	69.30 ± 3.23	70.83 ± 4.31	− 2.41 (.017)
Education	Elementary school	113 (55.4)	88 (49.2)	1.73 (.629)	59 (93.7)	76 (95.0)	4.18 (.243)
	Middle school	30 (14.7)	31 (17.3)		0 (0.0)	2 (2.5)	
	High school	47 (23.0)	44 (24.6)		2 (3.2)	0 (0.0)	
	College or higher	14 (6.9)	16 (8.9)		2 (3.2)	2 (2.5)	
Total household income (10,000 won)		1817.65 ± 1715.84	1704.94 ± 1455.17	0.69 (.492)	1217.56 ± 1228.36	1116.11 ± 1025.45	0.54 (.591)
Residence	Large city	40 (19.6)	65 (36.3)	26.04 (<.001)	16 (25.4)	30 (37.5)	4.35 (.114)
	Small city	42 (20.6)	53 (29.6)		10 (15.9)	17 (21.3)	
	Rural area	122 (59.8)	61 (34.1)		37 (58.7)	33 (41.3)	
Health status and behavior							
Subjective health status	Bad	39 (19.1)	32 (17.9)	0.13 (.937)	18 (28.6)	29 (36.3)	1.66 (.436)
	Moderate	96 (47.1)	87 (48.6)		30 (40.7)	38 (47.5)	
	Good	69 (33.8)	60 (33.5)		15 (23.8)	13 (16.3)	
Number of chronic diseases		0.64 ± 0.84	0.77 ± 0.91	−1.38 (.169)	0.78 ± 0.89	1.11 ± 0.94	−2.16 (.032)
Body grip strength index (kg)		28.89 ± 5.35	29.41 ± 5.70	−0.90 (.363)	19.00 ± 4.01	18.13 ± 3.82	1.28 (.202)
Depression score		3.19 ± 2.70	3.31 ± 2.80	−0.43 (.379)	4.02 ± 2.80	5.31 ± 2.46	−2.91 (.004)
Regular exercise (Yes)		41 (20.1)	63 (35.2)	10.99 (.001)	6 (9.5)	10 (12.5)	0.31 (.575)
Current smoking (Yes)		76 (37.3)	66 (36.9)	0.01 (.938)	0 (0.0)	2 (2.5)	1.60 (.206)
Current drinking (Yes)		123 (60.3)	110 (61.5)	0.05 (.817)	9 (14.3)	11 (13.8)	0.01 (.927)
Social support system/Interaction							
Live alone (Yes)		5 (2.5)	12 (6.7)	4.07 (.044)	31 (49.2)	32 (40.0)	1.21 (.271)
Existence of spouse (Yes)		193 (94.6)	165 (92.2)	0.92 (.337)	20 (31.7)	34 (42.5)	1.73 (.188)
Number of meetings with friends	No meetings	2 (1.0)	6 (3.4)	3.79 (.285)	3 (4.8)	2 (2.5)	0.64 (.886)
	1–6 times a year	9 (4.4)	10 (5.6)		3 (4.8)	3 (3.8)	
	1–4 times a month	108 (52.9)	99 (55.3)		28 (44.4)	37 (46.3)	
	Almost daily	85 (41.7)	64 (35.8)		29 (46.0)	38 (47.5)	
Participation in regular meetings (Yes)		169 (82.8)	131 (73.2)	5.24 (.022)	39 (61.9)	48 (60.0)	0.05 (.817)
Number of leisure activities		1.12 ± 2.04	1.09 ± 2.82	0.11 (.912)	0.52 ± 0.95	1.03 ± 1.90	−2.06 (.042)
Time spent on self-development		0.39 ± 2.25	0.50 ± 3.26	−0.37 (.711)	0.00 ± 0.00	0.94 ± 4.04	−2.08 (.041)

(*N* = 526)

**Fig. 1:**
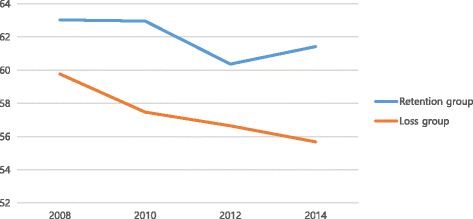
Patterns of quality of life according to job retention

**Fig. 2:**
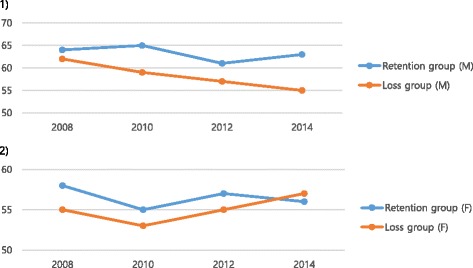
Patterns of quality of life according to job retention for men (1) and women (2)

**Fig. 3:**
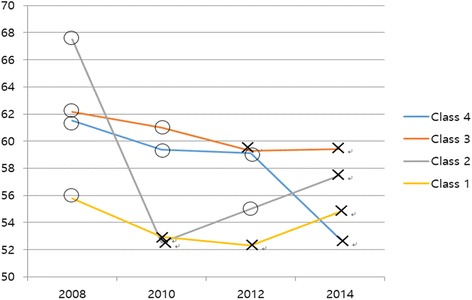
Relationship between point of job loss and quality of life of groups of respondents who had lost their jobs. O: Job retention; X: Job loss. Class 1: 2008 job retention, 2008–2010 Job lost groups. Class 2: 2008–2010 job lost, 2010–2012 job retention, and 2012–2014 job lost groups. Class 3: 2008–2010 job retention, 2010–2012 job lost groups. Class 4: 2008–2012 job retention, 2012–2014 job lost groups

Table 2 shows that the overall QOL of the job retention group and job loss group differed, at 63.03 and 59.77, respectively, in 2008 (*t* = 2.21, *p* = .027). This gradually declined over time in both groups, although the difference remained until 2014 (Fig. 1). However, there was no difference when the respondents’ data from 2008 were divided into the responses from men (*t* = 1.44, *p* = .150) and women (*t* = 1.27, *p* = .206) (Fig. 2). The relationship between the point at which a respondent lost his or her job and his or her QOL followed a similar pattern after the loss of a job in for all of Classes 1–4: after each respondent lost a job, that person’s QOL declined (Fig. 3). In men, there was no difference in QOL in 2008 when both groups had jobs, but there was a difference of about 6 points in 2010 (*t* = 0.12, *p* < .001), 4 points in 2012 (*t* = 2.45, *p* = .015), and 8 points in 2014 (*t* = 5.08, *p* < .001). However, for women, there was no statistically significant difference in QOL from 2008 to 2014 for both the job retention group and job loss group (Table 2).

**Table 2:** Changes and differences in quality of life according to year

Variables	Total (*N* = 526)			Men (*N* = 383)			Women (*N* = 143)		
(Year)	Retention (*N* = 267)	Loss (*N* = 259)	Difference	Retention (*N* = 204)	Loss (*N* = 179)	Difference	Retention (*N* = 63)	Loss (*N* = 80)	Difference
	Mean ±SD	Mean ±SD	t (*p)*	Mean±SD	Mean± SD	t (*p)*	Mean ±SD	Mean±SD	t (*p)*
2008	63.03 ± 16.32	59.77 ± 17.52	2.21 (.027)	64.41 ± 15.89	62.01 ± 16.64	1.44 (.150)	58.57 ± 17.03	54.75 ± 18.49	1.27 (.206)
2010	62.96 ± 16.31	57.47 ± 17.30	3.74 (<.001)	65.54 ± 14.80	59.49 ± 16.31	0.12 (<.001)	54.60 ± 18.21	52.91 ± 18.68	0.54 (.589)
2012	60.37 ± 14.89	56.65 ± 18.91	2.50 (.013)	61.42 ± 14.70	57.19 ± 18.53	2.45 (.015)	56.98 ± 15.10	55.44 ± 19.79	0.53 (.600)
2014	61.42 ± 14.34	55.68 ± 16.82	4.21 (<.001)	63.09 ± 13.00	55.08 ± 17.20	5.08 (<.001)	56.03 ± 17.00	57.00 ± 15.94	−0.35 (.141)

Linear mixed models were used to confirm the effects of employment on QOL. The VIF value for multicollinearity confirmed before the final model analysis was 1.15~ 3.12, indicating that there was no problem with multicollinearity. Table 3 shows QOL according to the presence or absence of work and over time. In the case of men, QOL over time was significantly higher for the job retention group than the job loss group (β = 7.751, *p* < .001). In terms of comparing changes in QOL over time, QOL in 2010 was no different from that of 2008 in both groups; however, QOL in 2012 (β = − 3.805, *p* = .003) and 2014 (β = − 4.254, p < .001) was significantly lower. In addition, the groups of those who had retained or lost their job showed significant differences in how their QOL changed over time; QOL was significantly lower in 2010 (β = − 3.570, *p* = .027) and 2014 (β = − 5.604, *p* = .003), confirming that time and work had an impact on it. The factors that affected QOL were total household income in the previous year, residential area, and depression score; QOL was higher when a respondent’s total household income in the previous year was high (β = 0.001, *p* = .002). In terms of residential area, QOL was significantly lower for those living in large cities than those in rural areas (β = − 4.921, *p* < .001). Furthermore, the more depressed individuals were, the lower their QOL was (β = − 0.750, p < .001).

**Table 3:** The quality of life according to job and time

	Men (N = 383)			Women (N = 143)		
Variables	B	*p*	95% CI	B	*p*	95% CI
Age	−0.186	.078	− 0.391~ 0.020	−0.231	.263	−0.637~ 0.174
Education (ref: College)						
Elementary	−6.308	<.001	−9.670~ − 2.946	− 7.735	.097	−16.884~ 1.413
Middle	−4.198	.022	−7.783~ − 0.613	−13.727	.055	−28.337~ 0.884
High school	−5.513	.001	−8.758~ − 2.267	−4.150	.587	−19.161~ 10.862
Total household income (10,000 won)	0.001	.002	0.000~ 0.001	0.004	<.001	0.003~ 0.006
Residence (ref: Rural area)						
Large city	− 4.921	<.001	−7.115~ − 2.726	− 4.167	.030	− 7.937~ − 0.395
Small city	1.272	.253	−0.910~ 3.454	−3.043	.141	−7.093~ 1.007
Subjective health status (ref: Good)						
Bad	−2.676	.040	−5.230~ − 0.121	−1.768	.475	−6.623~ 3.086
Moderate	0.179	.849	−1.647~ 2.006	−2.247	.287	−6.390~ 1.896
Number of chronic diseases	−0.099	.843	−1.079~ 0.881	− 0.986	.259	−2.702~ 0.730
Body grip index (kg)	0.112	.164	−0.046~ 0.271	0.134	.502	−0.257~ 0.524
Depression score	−0.750	<.001	−1.063~ − 0.437	− 1.232	<.001	− 1.872~ − 0.592
Regular exercise: No (ref: Yes)	−0.412	.679	−2.367~ 1.543	− 0.322	.889	−4.871~ 4.227
Current smoking: No (ref: Yes)	2.554	.004	0.833~ 4.275	−3.302	.607	−15.903~ 9.299
Current drinking: No (ref: Yes)	0.713	.397	−0.939~ 2.364	−3.386	.165	−8.172~ 1.400
Live alone: No (ref: Yes)	−1.562	.527	−6.409~ 3.285	− 6.372	.005	−10.837~ − 1.907
Existence of spouse: No (ref: Yes)	2.949	.324	−2.911~ 8.808	−4.250	.051	−8.510~ 0.010
Number of meetings with close friends (ref: Almost daily)						
No meetings	−5.256	.261	−12.609~ 2.098	−9.104	.125	−19.815~ 1.608
1–6 time a year	−6.543	.003	−11.338~ − 1.748	−7.337	.118	−15.874~ 1.200
1–4 times a month	−1.344	.391	−3.472~ 0.784	−2.901	.227	−6.813~ 1.012
Participation in regular meetings: No (ref: Yes)	−2.645	.011	−4.689~ − 0.601	−2.731	.089	−5.878~ 0.416
Number of leisure activities	0.163	.360	−0.187~ 0.513	0.387	.430	−0.576~ 1.350
Time spent on self-development	0.332	.038	0.018~ 0.647	0.269	.301	−0.242~ 0.781
Group job retention (ref: job loss)	7.751	<.001	4.464~ 10.808	−4.651	.128	−10.660~ 1.357
Time (yr): 2010 (ref time: 2008)	−0.911	.998	−3.582~ 1.759	−2.744	.479	−7.432~ 1.945
Time (yr): 2012 (ref time: 2008)	−3.805	.003	−6.573~ − 1.037	−1.240	.239	−5.955~ 3.475
Time (yr): 2014 (ref time: 2008)	−4.254	<.001	−6.929~ − 1.579	−0.642	.362	−5.432~ 4.148
Group*time 2010 (ref group*time 2008)	−3.570	.027	−6.741~ 0.399	2.289	.834	−3.135~ 7.712
Group*time 2012 (ref group*time 2008)	−1.749	.351	−5.434~ 1.935	2.224	.474	−3.896~ 8.345
Group*time 2014 (ref group*time 2008)	−5.604	.003	−9.291~ 1.912	4.790	.143	−1.641~ 11.221

ǂ 95% CI: Confidence Interval

In the case of women, being employed, the passage of time, and interactions of group and time were not significant. The factors that affected QOL were the same as those of men, i.e., total household income in the previous year, residential area, and depression score; QOL was higher when total household income in the previous year was high (β = 0.004, *p* < .001). In terms of residential area, QOL was significantly lower for those living in large cities than those in rural areas (β = − 4.067, *p* = .030). The more depressed individuals were, the lower their QOL was (β = − 1.232, p < .001).

Aside from the significant factors described above, the factors affecting the QOL of men included current smoking (β = 2.554, *p* = .004), social interaction in terms of number of meetings with close friends (β = − 6.543, *p* = .003), participation in regular meetings (β = − 2.645, *p* = .011), and time spent on self-development (β = 0.332, *p* = .038). On the other hand, living alone was identified as a significant factor affecting QOL of for women (β = − 6.372, *p* = .005) (Table 3).

## Discussion

The purpose of this study was to investigate the effects of employment on the QOL of older adults during the period of 2008–2014. The results show that work was an important factor in QOL. For men, the QOL of the job retention group was higher than that of the job loss group, which supports the finding of many previous studies that QOL is higher among the elderly who are employed [15, 17, 18, 26]. Furthermore, in men, QOL interacted with time, such that change in QOL over time was greater in the job loss group than in the job retention group. However, in the case of women, job retention did not affect QOL. One reason for this trend might be a lack of statistical power due to the relatively small number of women in the study; another possible reason is that Korean society encourages women to devote themselves to their family, rather than take advantage of opportunities for education and social participation. The characteristics of the elderly women in this study were that their average age was 70 years, they had less employment experience, and had lived for their family rather than for themselves. Therefore, it is necessary to study in more detail, the relationship between work and the QOL of elderly women workers in South Korea using large samples.

Other factors influencing the QOL of the elderly were total household income and depression. The results of this study show that the higher a respondent’s total household income, the higher his or her QOL; this supports previous studies indicating that economic status affects QOL [27, 28]. However, the relationships among depression, occupation, and QOL for older adults are not yet clear. The results of previous studies using HRS and SHARE data to identify depression in the unemployed in the United States and Europe revealed a higher incidence of depression in the unemployed [14], and the more depressed someone is, the lower his or her QOL [26]. Previous studies also indicated that depression scores were an important factor in QOL. Such results suggest that older adults in South Korea, where there are relatively fewer employment and welfare systems for the elderly than that in the United States and Europe [9], can be expected to experience more severe depression. Further research is required to confirm the relationships between occupation status, depression, and QOL.

In men, social support systems such as residential area and participation in regular meetings differed between groups. Traditional rural Korean communities are often engaged in community-based farming and leisure activities; the results of this study show that older adults living in rural areas find it easier to keep their jobs and have a higher QOL. Consequently, the results suggest that in large cities where family ties are weakened, a lower QOL is related to elderly people engaging less often in social interactions. This is supported by the fact that previous studies have indicated that having more friends and participating more in physical leisure activities and volunteer services improves QOL and lowers the mortality rate [29–31]. Therefore, it is necessary to implement policies that recognize and support employment as an important factor affecting QOL for older adults, as a means of interacting and communicating with many people, as well as a means of earning a livelihood. The results of this study also show that the QOL for elderly men was influenced more by whether they currently smoked than by the number of chronic diseases they suffered from, the amount of regular exercise they performed, and current alcohol consumption. Therefore, considering the low QOL of elderly male smokers, a follow-up study is required to assess how smoking is related to job retention and job loss, which is known to be either a direct or an indirect factor in physical and psychological health status. In addition, this study found that living alone affected elderly women’s QOL more than employment. Elderly women may experience poor QOL if they lack time, face discomfort when taking care of their retired husbands, and if they are struggling with economic difficulties or physical pain [32, 33]. Therefore, future research should investigate the effects of QOL, family characteristics, and physical and psychological factors on elderly women.

In 2004, the government initiated the “Elderly Jobs Project” for older adults to prepare them for the problems associated with an aged society. However, while this initiative increased the number of simple jobs to support one’s livelihood for older adults, it did not guarantee a reliable income for them, instead encouraging leisure activities through social participation and health promotion. To compensate for this, in 2015, the name of this initiative was changed to “Senior Citizenship and Social Activity Support Project,” and the focus was shifted to volunteer work and employment; the revised project was then implemented. However, there are restrictions on older adults who want to work participating in the Senior Citizenship and Social Activity Support Project if they receive benefits under the National Basic Livelihood Security Act, Medicare, or the National Health Insurance Workplace [34].

In 2016, South Korea had the second-longest average annual working time in the OECD, at 2069 h per worker [35]. Those who work such long hours have poor QOL, although those who do not work also had lower QOL. To solve this problem and ensure a stable income for older adults, flexible work arrangements are needed.

This study has some limitations. First, the patterns of QOL according to the employment status of 526 older adults between 2008 and 2014 were studied; however, some prospective participants were excluded because we could not confirm that they had experienced a change in employment. Thus, it may not be possible to generalize our results to the entire population of older adults may be limited. Second, the number of women included in the sample was small, and there is lack of statistical power to explore the relationship between job status and the QOL of elderly women. In addition, this study involved a self-report questionnaire, so the potential for biased reporting exists. Due to this being a secondary data analysis, data on the amount and duration of current smoking and drinking were not available, nor were data on food intake. Finally, our understanding of the circumstances under which older adults lose their jobs was limited because we did not consider the characteristics of each job. However, this study is meaningful as a longitudinal study in which older adults provided specific data on their QOL associated with the retention and loss of employment. In addition, by suggesting factors that affect QOL, the results can be utilized to expand employment of the elderly in the future.

## Conclusion

This study was a secondary data analysis that investigated the effect of job retention and loss on the QOL of the elderly using KLoSA data from 2008 to 2014. This study showed the effects of work on the QOL of older adults, whether they were currently or formerly employed. It is a meaningful finding that employment was an important factor for QOL.

Based on the results of this study, we propose the following measures. First, to improve the QOL of older adults, tailored programs are needed to understand the characteristics of the elderly, such as gender and cultural characteristics, and to create jobs that are suitable for them. For older men working with others, entertainment, self-development, and volunteering are necessary to encourage them spend time with others outside of the home. Second, to generalize the relationship between job retention and the QOL of older adults, studies on elderly women workers need to be repeated with adequate sample sizes. Third, studies should continue to clarify the relationship between occupation and health-related factors such as depression and smoking. Finally, in future research, it is necessary to confirm changes in QOL over time of young employed women, who grew up in a culture different from those of elderly women and for whom the meaning of having a job might be different.

## Abbreviations

ELSA: English Longitudinal Survey of Aging
HRS: Health and Retirement Survey
KLoSA: Korean Longitudinal Study of Aging
QOL: Quality of life
SHARE: Survey of Health, Aging and Retirement in Europe
